# How to identify insular epilepsy

**DOI:** 10.1007/s00415-022-11093-z

**Published:** 2022-04-13

**Authors:** Andreas Schulze-Bonhage, Eva Martinez Lizana

**Affiliations:** Epilepsy Center, University Medical Center, University of Freiburg, Freiburg, Germany

Dear Sirs,

Reply to the letter by Hagiwan and Isnard.

We thank Dr. Hagiwan and Dr. Isnard for their attention to our publication on ictal semiology of epileptic seizures with insulo-opercular generis [4].

Dr. Hagiwan and Dr. Isnard correctly state that this study compared the semiology of insular epilepsy with mesiotemporal epilepsy and did not address patients with temporo-polar epilepsy or other brain regions with different spectra of semiological signs and symptoms. We are, however, surprised that the authors do not consider the study relevant to the problem of “temporal plus” epilepsies as insular co-involvement in seizure generation is relevant question in this topic and as semiological characteristics elaborated by statistical comparison of classical mTLE and epilepsies of insular origin can be useful to point to a possible additional or even only seizure generator in the insular cortex. For this distinction, our study used strictly separated groups.

We furthermore do not agree that there is no longer a clinical problem in differentiating mTLE from patients with insular seizure origin. Notably, Isnard himself [1] has published a series of 50 patients undergoing implantation of temporo-insular SEEG electrodes for focus localization, of whom only 5 (10%) turned out to have an insular electrographic seizure origin. Obviously, one aim of a better differential characterization of insular and mesiotemporal seizure semiology is to avoid invasive diagnostics in those 90% of patients in whom there was no insular involvement in seizure generation.

Moreover, there is a vast literature on postoperative outcome reports, showing that seizure freedom is only achieved in 60–65% of patients in whom mesiotemporal epilepsy surgery is performed [e.g., 6, 7], including selective approaches like LITT [2], clearly showing that there is a need to improve patient selection for selective mesiotemporal interventions and to differentiate this group from seizure origin in other brain regions with similar electroclinical features. It is a misunderstanding of our publication that 100% of patients with mesiotemporal seizure origin when operated become seizure free—this gold standard of postoperative seizure freedom for correct localization of the epileptogenic zone was an inclusion criterion for the comparator group with mTLE rather than an outcome report.

The authors furthermore point out that several signs which may be typical for insulo-opercular origin were not reported as discriminating factors between mTLE and insular epilepsy. Our study applied statistical methods to distinction of mTLE and insular epilepsy, and several signs considered typical for insular seizures nevertheless occur only rarely and thus did not contribute significantly to a discrimination at a group level.

Hagiwan & Isnard furthermore criticize that only part of the patients reported had undergone intracranial SEEG recordings to establish an insular origin of their seizures.

This is correct; in our series, only non-lesional patients based on advanced MR imaging had obligatory SEEG exploration, whereas patients with circumscribed lesions like insular cavernomas or tumors frequently underwent direct lesionectomy as surgical treatment.

SEEG suffers from a considerable undersampling of the insular cortex, particularly when performed with a lateral insertional approach, with the inherent risk of false localization of a presumed seizure onset zone based on the first contact displaying an ictal EEG pattern. SEEG furthermore carries an inherent risk of bleeding even in groups using latest technologies for electrode implantation [3, 5], and particularly so in the insular region. Third, there is so far no evidence that SEEG performed in lesional cases contributes to an improved delineation of the epileptogenic area which is reflected in postsurgical outcome, and in our view, the individual decision to perform SEEG recordings has to balance inherent risks of this invasive approach with the additional information that can be expected in clear lesional cases.

We completely agree with Hagiwan and Isnard with regard to the importance of a distinction between the symptomatogenic zone and the zone of seizure onset, which had been established decades ago, and that motor symptoms in insular epilepsy to a large degree reflect propagation of epileptic activity to frontal regions—as discussed in the publication. Nevertheless, semiological analyses have provided knowledge relevant for the planning of epilepsy surgery as characteristic propagation patterns reflect both, the eloquence of brain regions and of their connectivity. An example of the role of connectivity was the finding of a significantly higher frequency of bilateral sensorimotor manifestations in insular epilepsy compared to seizures of temporal origin. In our view, detailed semiological analyses comparing specified brain areas of seizure origin and the integration of this information into multimodal electrophysiological and imaging data will remain an important tool to improve presurgical workup and surgical planning.

Few studies on the manifestation of insular epilepsy have been based on patients rendered seizure free by selective surgery of insular subregions. A considerable subgroup of our patients was operated with selective lesionectomies or topectomies in the insular region, and—as discussed in the publication—a joint multicenter analysis of data from centers in whom superselective insular resections are performed will be an important next step to go. We are very open to collaborate on this with other groups, including those from Fukuoka and Lyon.

Sincerely,

Andreas Schulze-Bonhage and Eva Martinez-Lizana.

Epilepsy Center, University Medical Center—University of Freiburg, Germany.
